# Electric Ozonation in Neuralgia

**Published:** 1902-09

**Authors:** G. Lenox Curtis

**Affiliations:** New York


### ELECTRIC OZONATION IN NEURALGIA.[1]

BY G. LENOX CURTIS, M.D., NEW YORK.[2]

THE term neuralgia may be applied to all pains found in animal tissue that may be regarded as being nearly or quite in a perfect physical condition, especially if the pains have become chronic. To designate the locality of the cause of the pain, which may or may not be in the region of the pain, and leaving out what may be regarded as functional disturbance, we may mention, for illustration of my subject, facial neuralgia, gastralgia, myalgia, as of the pain in myelitis and pyelitis, all of which are only different phases of disturbances called neuralgia. The locality of the keenest pains may be in the parts suggested by those names, or they may be at greater or less distance. Neuralgia in and about the face and mouth is generally easily determined, but there are causes where there seem to be complications.

Other forms of pain, such as gout, rheumatism, sciatica, and lumbago, may all come under the general term of affections of the

---

[1] Read before the Section on Stomatology of the American Medical Association, at Saratoga Springs, June, 1902.

[2] Member of the American Medical Association, New York Medical Association, Harlem Medical Association, Medical Association of the Greater City of New York, Consultant to the Red Cross Hospital.

nerves, and may all be treated in connection. But this would open a wider field than I desire to discuss at this time.

The pain about a tooth in one jaw may cause pain in the opposite jaw. It is well known that a defective lower side tooth often causes pain in the upper tooth. But it may be said that in cases of pulpitis, gingivitis, periostitis, and pericementitis the pains are found in the parts involved. As these latter forms of neuralgia are probably the ones that will most interest this section of medicine, I will confine my remarks mainly to them. My aim is to call the attention to the power of electric ozonation; its effect upon this disease I regard as a comparatively new phase of practice in medicine.

After three years of experimental work, I called the attention of the medical profession, in October, 1901, to it in a paper read before the Academy of Medicine, in New York.[1] In that paper the plan by which ozone may be properly made, and at the same time enable the practitioner to force it directly into and through the affected parts, was clearly set forth. I also explained its effects and gave report of cases in which the stimulating and ozonizing speedily re-established the normal functions. When nerve-force and proper nutrition are established, and equilibrium is re-established, a high condition of health is the result. I do not wish to be understood as saying that by electric ozonation a normal physical condition can be re-established in a diseased organ, such as follows the loss of tubules in chronic nephritis, abscesses of the lungs and liver, or a cicatrix in a nerve-trunk following a traumatic lesion or calcific deposit in the pulp of a tooth. But I do imply that by electric ozonation nerve-force and circulation can be sufficiently re-established in the parts to lead to health, and the parts be left in the best condition possible under the circumstances.

The mechanism that will make this remarkable element is a system of coils of fine wire, so arranged as to change the quality of the current of electricity from the street into the wonderful therapeutic agent.

One of the changes in the electric current effected by passing through the machine is increase of voltage, while at the same time the amperage is reduced, thus causing a high tension current. The

---

[1] This paper was published in the New York Medical Journal of January 18 and February 1, 1902.

capacity of this machine is one million volts and about one-fiftieth of an ampere, capable of producing a large amount of ozone. The higher the voltage, and the lower the amperage, the less is the degree of shock experienced by the patient. It re-establishes nervous functional activity, thus stimulating tissue repair.

The current from this machine, which seemingly has but a single pole, passes through the body and then escapes into the atmosphere, which may be regarded as a negative pole.

Among the advantages of this machine is easy handling; that is, it is easily carried about when travelling. The fact that it may be used wherever there is an incandescent current, without its being affected by conditions of the weather, is an important virtue.

Removing the cause of neuralgia generally stops pain,—for illustration, the removing of a pulp-stone, necrosed bone, an irritating filling, gas in pulp-chamber, or alveolar abscess pressure,—but there are cases that require the element of greater length of time before unanimity is established, and electric ozonation tends to shorten this time.

Among other disturbances of the nervous system that may be mentioned are those that cause hemiplegia and catalepsy. But association with other disturbers sometimes embarrasses diagnosis. I recall a case of hemiplegia, supposed to be from brain lesion, that was completely dissipated after the removal of an alveolar abscess. I also recall another case of catalepsy from the same cause. Slight irritation may not cause neuralgia when vital force is at par, but when vitality is low neuralgia in most any part of the system may continue, and lead to neurasthenia.

While investigating the cause of pain, for the purpose of giving relief, the practitioner generally believes that the pain is the result of a lesion. Should lesion not be found, I think that the surgeon should not operate. I think that the practitioner should never operate for relief until a careful and exhaustive examination of the patient's system is made and he has ascertained the full conditions of health. This examination may show that the patient is in a very debilitated condition, and the vitality so low that there is not sufficient supply of nerve-force for a high condition of health. Under such circumstances, the overworked and inflamed nervous system needs assistance, and, as pain is nature's voice calling for help, it may be regarded a blessing in disguise. To illustrate the importance of making careful diagnosis, and to show how we may help nature, I will present two cases, recently in my practice, one in which a lesion was determined, the other in which a lack of vital force was the cause of the pain. Mr. I., aged eighty, had suffered for twenty-five years from facial neuralgia of a very acute character. All his teeth, one after another, had been extracted, without giving any abatement of the excruciating agony. Under these conditions his health gradually failed, and the paroxysms of pain became more and more intense until continued agony made his life hardly worth preserving. This was his condition when brought to me by his physician for consultation regarding advisability of resection of the lower dental nerve. Upon a careful examination, I found that his nervous system was in a very feeble condition, and with its present capabilities would not generate enough nerve-force to furnish half the vitality necessary for even a moderate condition of health.

It was clear to me that his nervous system must be awakened and made more vigorous before improvement could be seen. I suggested a course of ozonation, and the advice was acted upon. After two weeks of daily ozonation his general health had improved to a marked degree, and with this change rapidly came the gratifying result,—entire freedom from pain. This ozonation treatment was continued two weeks longer, when sound health was firmly established.

This highly satisfactory condition lasted a year, when the death of a dear member of his family caused him great sorrow, and necessitated a long and fatiguing journey by railroad. The grief and journey combined so exhausted his vitality that neuralgic pain was again felt in full force. He came to me again for treatment, was treated by the same method, and was again restored to vigorous health. He now continues to live and enjoy life free from pain.

Another case was that of Mrs. E., who had, for twelve years before I saw her, been subject to long periods of suffering from neuralgia in one side of the face. She had, one at a time, all the molars extracted without gaining relief. During the examination I asked her what had been the condition of her general health. She replied that her "health was good," but her nervous, anxious expression contradicted her assertion and showed clearly the irritable state of her system. I continued my examination, and concluded the cause to be pulp-stones in the upper bicuspids of the affected side, and advised the removal of the pulps, or possibly extraction of the teeth. I found her vitality very low, none of the functions of her organs performing regularly, thus showing that a patient's word cannot always be relied upon in such matters. In no sense was she a well woman.

She would not consent to surgical treatment that seemed proper, but after the ozone treatment was suggested she concluded to accept it. After the second treatment all signs of pain in the parts disappeared, and after a month's treatment her general health was seemingly entirely restored, and for two months she was free from pain. But later, contracting a violent cold, and the bicuspids becoming troublesome with slight paroxysms of pain, she again came to me for relief. After several ozone treatments all pain disappeared. When last I saw her she was in good health, and had no returns of the neuralgia. I believe, however, that until the pulp-stones are removed she will occasionally have a recurrence of the pains when her vitality runs low. I may mention that previous to the time that she came to me she was considering the resection of the gasserian ganglion.

I have observed similar results in other cases.

